# Randomized controlled clinical study on Yiqi Liangxue Shengji prescription for intervention cardiac function of acute myocardial infarction with ischemia-reperfusion injury

**DOI:** 10.1097/MD.0000000000024944

**Published:** 2021-03-12

**Authors:** Cong Chen, JunYan Xia, Ruli Feng, Jie Wan, Kun Zhou, Qian Lin, Dong Li

**Affiliations:** a Department of Cardiology, Dongzhimen Hospital Affiliated to Beijing University of Chinese Medicine; b Department of Cardiology, Dongfang Hospital Affiliated to Beijing University of Chinese Medicine, Beijing, China.

**Keywords:** acute myocardial infarction, myocardial ischemia-reperfusion injury, percutaneous coronary intervention, randomized controlled trial, Yiqi Liangxue Shengji prescription

## Abstract

**Introduction:**

The morbidity and mortality of acute myocardial infarction patients still remains high after percutaneous coronary intervention (PCI). Myocardial ischemia-reperfusion (MIR) injury is one of the important reasons. Although the phenomenon of MIR injury can paradoxically reduce the beneficial effects of myocardial reperfusion, there currently remains no effective therapeutic agent for preventing MIR. Previous studies have shown that Yiqi Liangxue Shengji prescription (YLS) is effective in improving clinical symptoms and ameliorating the major adverse cardiovascular events of coronary heart disease patients undergoing PCI. This study aims to evaluate the effectiveness and safety of YLS in patients with acute myocardial infarction (AMI) after PCI.

**Methods:**

This study is a randomized, double-blinded, placebo-controlled, single-central clinical trial. A total of 140 participants are randomly allocated to 2 groups: the intervention group and the placebo group. Based on routine medications, the intervention group will be treated with YLS and the placebo group will be treated with YLS placebo. All participants will receive a 8-week treatment and then be followed up for another 12 months. The primary outcome measures are N terminal pro B type natriuretic peptide (NT-proBNP) and left ventricular ejection fraction. Secondary outcomes are plasma levels of microRNA-145, plasma cardiac enzyme, and Troponin I levels in blood samples, changes in ST-segment in ECG, Seattle Angina Questionnaire, the efficacy of angina symptoms, and occurrence of major adverse cardiac events. All the data will be recorded in case report forms and analyzed by SPSS V.17.0.

**Discussion:**

The trial will investigate whether the postoperative administration of YLS in patients with AMI after PCI will improve cardiac function. And it explores microRNAs (miRNA)-145 as detection of blood-based biomarkers for AMI by evaluating the relation between miRNAs in plasma and cardiac function.

**Trial registration:**

Chinese Clinical Trials Registry identifier ChiCTR2000038816. Registered on October 10, 2020.

**Strengths and limitations of this study**

- This is the first randomized, double-blinded, placebo-controlled, single-central clinical trial to explore the effectiveness and safety of Yiqi Liangxue Shengji prescription for the treatment of patients with acute myocardial infarction after percutaneous coronary intervention.
- Additive or synergistic cardioprotective effects of multiple, instead of single, cardioprotective agents will be better cardioprotective strategies to reduce myocardial infarction.
- This pilot study explores that in the setting of microRNAs -145 will be a novel biomarker for the diagnosis and prognosis of cardiovascular diseases and promising therapeutic strategy.
- This study is limited by the lack of clinical endpoints and the small sample size. Moreover, the trial will be undertaken in China, it is uncertain whether the similar effects are available to other ethnic groups and regions.

## 1 Introduction

Despite recent progress in the diagnosis and treatment of cardiovascular diseases, coronary heart disease (CHD) represents the leading cause of death and disability worldwide.^[1,2]^ According to the WHO, CHD had caused 7,254,000 deaths worldwide (12.8% of all deaths) in 2008.^[3]^ After an acute myocardial infarction (AMI), timely and successful myocardial reperfusion with the use of thrombolytic therapy or percutaneous coronary intervention (PCI) is the most effective strategy for reducing the size of a myocardial infarct and improving the clinical outcome. However according to report, timely reperfusion by primary percutaneous coronary intervention (PPCI) and secondary preventative therapies still bring significant morbidity and mortality, with 7% death and 22% heart failure at 1 year.^[4]^ The process of restoring blood flow to the ischemic myocardium, however, can induce injury. The phenomenon of myocardial ischemia-reperfusion (MIR) injury can paradoxically reduce the beneficial effects of myocardial reperfusion.^[5]^ MIR often causes in further myocardial injury and cardiomyocyte death, which occurs in up to 50% of reperfused ST-segment elevation myocardial infarction patients, and its presence portends to a worse prognosis.^[6,7]^ And growing evidence from experimental studies MIR greatly contributes to the final infarct size.^[8,9]^ Although the process of myocardial reperfusion with advances in PCI technology that can timely and effective reperfuse coronary blood flow to ischemic myocardium or with antiplatelet and antithrombotic agents for maintaining the patency of the infarct-related coronary artery only reduce MIR,^[3]^ there currently remains no effective therapeutic agent for preventing MIR.^[4]^

Chinese herbal medicine (CHM), especially combined herbal formulations, has been widely used in traditional Chinese medicine for the treatment of myocardial infarction for hundreds of years and has attracted increasing attention in drug research.^[10]^ In the theory of Traditional Chinese Medicine (TCM), the collocation of monarch herbs and minister herbs is assembled according to the pathogenesis of patients with further prescription to adapt to the different pathogenesis of MIR. In the diagnosis of MIR, “blood stasis and heat syndrome” is an important syndrome based on the viewpoint of TCM theory and our previous clinical practice.^[11]^ TCM practitioners attach importance to preventing disease before it arises and controlling the development of existing disease. And, there are some special advantages for TCM in ameliorating myocardial ischemia-reperfusion injury.^[10]^

Yiqi Liangxue Shengji prescription (YLS) is a based on the TCM theory of “invigorating qi, activating blood and clearing up the heat of blood” for the prevention of MIR in China. Clinical trials have shown that YLS is effective in improving clinical symptoms and ameliorating the major adverse cardiovascular events (MACE) of coronary heart disease patients undergoing percutaneous coronary intervention.^[12,13]^ YLS consists of 4 herbal medicines (Table 1), including Astragalus membranaceus (Huang Qi), Salvia miltiorrhiza (Dan Shen), Cortex Moutan (Mudan Pi), Lonicerae japonicae flos (Jinyin Hua). Among them, Radix Astragali and Salviae Miltiorrhizae are the principal pharmacologically active components. They exhibit superior anticardiac reperfusion injury action in animals, including restoring cardiac mechanisms, such as coronary blood flow, lipid peroxide content, superoxide dismutase activity, the release of inflammatory mediators, and so forth, to favorable levels after reperfusion.^[14,15]^ However, there is no clinical evidence for YLS in the treatment of MIR. Therefore, we designed a randomized, double-blinded, placebo-controlled, single-central clinical trial aiming to evaluate cardiac protection from Yiqi Liangxue Shengji prescription in patients with AMI after PCI.

**Table 1:** Components and dose of Yiqi Liangxue Shengji prescription.

Chinese name	English name	Pharmacological effects	Main active components	Weight (g/bag)	Reference
Huang Qi	Astragalus membranaceus	Anti-oxidation, regulation of mitophagy, anti-inflammation, immunomodulatory effects, anti-neurodegeneration,	Triterpene saponins (Astragalosides, Acetylastragaloside, AcetylastragalosideIsoastragaloside, Astramembrannin etc); Flavonoids (Isoflavonones, Isoflavans, Pterocarpans etc); Polysaccharides (Glucans, Heteropolysaccharide)	30	(Shan et al, 2019) (Liu et al, 2017)
Dan Shen	Salvia miltiorrhiza	Endothelial protective effects, proliferation and migration of VSMCs, improve microcirculatory, anticoagulant, antithrombotic, anti-inflammatory,	Diterpene chinone (tanshinone I–VI, cryptotanshinone, isotanshinone I–II, Danshenol A etc); phenolic acid (Danshensu, salvianolic acid A, salvianolic acid B, protocatechuic aldehyde, etc)	15	(Li et al, 2018)
Mudan Pi	Cortex Moutan	Anti-oxidative, anti-inflammatory, anti-tummor, vasodilatatory effects, improving blood circulation, inhibiting inflammation	paeonol, paeoniflorin, paeonoside, apiopaeonoside, oxypaeoniflorin, galloylpaeoniflorin, galloyloxypaeoniflorin, mudanpioside A, B, C, D, E, H, suffruticoside A, B, C, D, E, benzoyloxypaeoniflorin, benzoylpaeoniflorin and gallic acid, etc	10	(Wang et al, 2017)
Jinyin Hua	Lonicerae japonicae flos	Anti-inflammatory, bacteriostatic activity, antiviral activity, liver protective activity, anti-oxidative	Chlorogenic acids (Chlorogenic acid, Neochlorogenic acid, Isochlorogenic acid A, B, C, etc); Cinnamic acids (Caffeic acid, 3-O-Feruloylquinic acid etc); Benzoic acids (2,5-Dihydroxybenzoic acid-5-O-β-d-glucopyranoside)	10	(Li et al, 2019)

Li Y, Li W, Fu C, Song Y, and Fu Q (2019). Lonicerae japonicae flos and Lonicerae flos: a systematic review of ethnopharmacology, phytochemistry, and pharmacology. *Phytochem Rev*, 1–61. doi: 10.1007/s11101-019-09655-7.

Li ZM, Xu SW, and Liu PQ (2018). Salvia miltiorrhizaBurge (Danshen): a golden herbal medicine in cardiovascular therapeutics. *Acta Pharmacol Sin* 39, 802–824.

Liu P, Zhao H, and Luo Y (2017). Anti-aging implications of Astragalus Membranaceus (Huangqi): a well-known Chinese tonic. *Aging Dis* 8, 868–886.

Shan H, Zheng X, and Li M (2019). The effects of Astragalus Membranaceus active extracts on autophagy-related diseases. *Int J Mol Sci* 20(8). doi: 10.3390/ijms20081904.

Wang Z, He C, Peng Y, Chen F, and Xiao P (2017). Origins, phytochemistry, pharmacology, analytical methods, and safety of Cortex Moutan (Paeonia suffruticosa Andrew): a systematic review. *Molecules (Basel, Switzerland)* 22, 946.

## 2 Methods and design

### 2.1 Patient and Public Involvement

Patients and/or public were not involved in the different stages of the study (including the design and the recruitment phase). However, we intend to disseminate the main results to trial participants and will seek patient and public involvement in the development of an appropriate method of dissemination.

### 2.2 Study design

**Figure 1:**
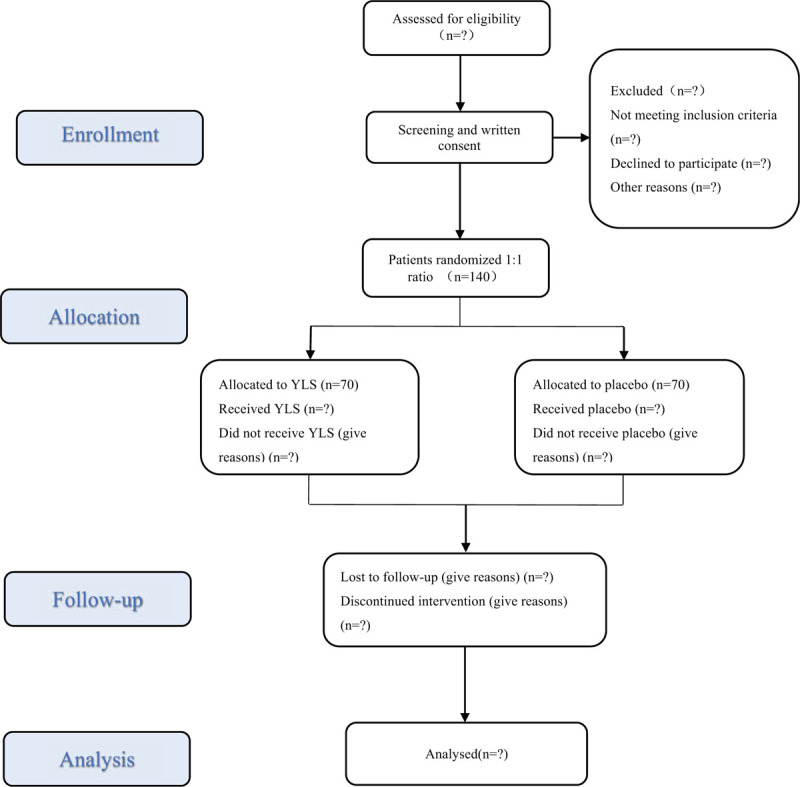
Study flowchart.

This is a single-central, randomized, double-blinded, placebo-controlled, parallel-group clinical trial. The study was registered at the Chinese Clinical Trial Registry on 10 October 2020 (ChiCTR2000038816). This study will be conducted in accordance with the principles of the Declaration of Helsinki and Good Clinical Practice guidelines. We will rigorously follow the latest Consolidated Standards of Reporting Trials (CONSORT 2017) for CHM recommendations,^[16]^ Standard Protocol Items: Recommendations for Interventional Trials and 2013 statement for herbal interventions.^[17]^ The study will recruit 140 AMI patients treated with PCI from inpatients and outpatients at the Dongfang Hospital, and has been approved by IRB of Dongfang Hospital Affiliated to Beijing University of Chinese Medicine (JDF-IRB-2020031102). All patients will provide written informed consent. The patient recruitment pathway is shown in Figure 1.

### 2.3 Overall objective

The trial will investigate whether the postoperative administration of YLS in AMI patients treated with PCI will improve cardiac function. And it explores microRNAs (miRNA)-145 as detection of blood-based biomarkers for AMI by evaluating the relation between miRNAs in plasma and cardiac function.

### 2.4 Patient inclusion and exclusion criteria

On arrival at the hospital, patients presenting AMI and treat with PCI will be screened for study eligibility and written informed consent will be obtained.^[18,19]^ Patients received standard secondary prevention treatment for coronary heart disease and Oral loading with 300 mg of Aspirin and clopidogrel hydrogen sulphate tablets (or 180 mg of ticagrelor) will be performed before surgery. Patient inclusion and exclusion criteria are listed in Table 2.

**Table 2:** Patient inclusion and exclusion criteria for the trial.

Inclusion criteria
1. 18 yr old ≤ age ≤ 80 yr old.
2. Provision of written informed consent by participants or surrogates voluntarily.
3. Clinical diagnosis of acute myocardial infarction (AMI) occurred within 12 h,^[22]^ and all participants must have been treated with PCI.
4. Patients are diagnosed as having a syndrome of deficiency of Qi and blood stasis according to TCM standards.^[23]^
Exclusion criteria
1. Severe valvular heart disease (aortic stenosis), severe psychoneurosis, climacteric syndrome, hyperthyroidism, cervical spondylosis, gallbladder heart syndrome, gastro-oesophageal reflux disease.
2. Uncontrolled hypertension with systolic blood pressure ≥180 mm Hg or diastolic blood pressure ≥110 mm Hg, severe cardiac insufficiency with Ejection Fraction< 35%, severe arrhythmia (fast atrial fibrillation, atrial flutter, paroxysmal ventricular tachycardia, atrioventricular block higher than second degree subtype II, complete bundle branch block).
3. Patients with severe primary diseases like heart, brain, liver, kidney, hemopoietic system-related diseases, and patients whose Alanine Aminotransferase or Aspartate Transaminase is higher than 1.5 times of the upper limit, patients with renal insufficiency (serum creatinine > 194.5 μmol/L) or insulin-dependent diabetes mellitus.
4. Patients with depression or anxiety.
5. Stroke or resuscitated sudden death in the past 6 mo.
6. Patients with malignant tumor.
7. Pregnant women or breast feeding women.
8. Hyperthyroidism with TSH levels more than 1.5 times upper limit of normal.
9. Increased bleeding risk (gastrointestinal bleeding, traumatic head injury, and bleeding diathesis).
10. Patients with allergic constitution or are allergic to many kinds of TCM herbs and ingredients of the study drug.
11. Participation in other clinical trials in last 1 mo.
12. Patients unable or unwilling to sign the informed consent form.

PCI = percutaneous coronary intervention, TCM = Traditional Chinese Medicine.

### 2.5 Removal, dropout, and termination criteria

Participants can voluntarily drop out at any time during the trial. Eligible subjects failing to complete the observation period presented in the trial will be considered as dropout cases regardless of the time and reason. Reasons for dropout will be recorded, and the last data recorded for these participants will be included in the data analysis. The trial will be suspended in a specific participant if serious adverse events (AEs) relevant to the YLS occur; the participant decides to join in another clinical research project in terms of cardiovascular diseases; the participant demonstrates hypersensitivity toward YLS, such as abnormal thirsty, stomach ache, and diarrhoea; the participant suffers from acute life-threatening disease. The whole research would be terminated in the following circumstances: masking of the randomization fails; unblinding rate exceeds 20% of the sample size; assessments of all follow-up are completed.

### 2.6 Randomization and masking

Consented patients will be randomized to receive either CHM or placebo using concealed random allocation from a computer-generated random numbers table through the Dongfang hospital Clinical Research Institute. Once randomized, the patient will receive postoperative administration of either YLS or placebo after reperfusion of the culprit artery, as soon as possible. The treatment allocation will be concealed from the patient, the interventional cardiologist, the blinded research staff collecting the data and clinical endpoints, and other staff analyzing the outcome data.

The eligible consented patient will then be randomized to receive either:

1. CHM treatment: YLS will be administered as a granule (1 bag at a time, 2 times per day, 8 weeks). This dosing regimen is identical to that used in the other trials.^[20]^ Furthermore, this dose was also found to be cardioprotective in a preclinical model using animal.^[21]^
2. Placebo control: Both YLS and placebo have the same outer packaging, color, shape, and flavor, so that neither the participant nor the investigator could recognize which group of intervention the participants are receiving before unblinding. After the treatment, the packaging will be returned to the investigators.

All patients will be transferred to the coronary care unit after the primary angiographic procedure. Intravenous glycoprotein IIb/IIIa inhibitor will be maintained for 18 to 36 hours after PCI. A loading dose of aspirin and P2Y12 inhibitors will be given before the procedure. An intravenous bolus of unfractionated heparin (100 U/kg) will be administrated right before the procedure to achieve therapeutic activated clotting time. Dual-anti-platelet therapy will be maintained during the deferred period and for at least 1 year after PCI. The peri-procedural treatment is in accordance with the Chinese guidelines for the management of AMI.^[22]^

### 2.7 Sample size calculation

The formula used to calculate the sample size is as follows, which is based on superiority clinical trial hypothesis test sample size estimation.^[23]^ The sample size was calculated on the basis of expected reduction in NT-proBNP. One previous study suggested that the reducing value for NT-proBNP level in AMI patient treatment with PCI is 32%. Therefore, we assume the reduction in NT-proBNP level as 58% in this study. Given a type I error rate of α = 0.05, type II error rate of β = 0.2, Uα(0.05)=1.65, Uβ (0.2) =1.28. The sample size for 1 arm needs to be 63, resulting in n = 2 × 63 = 126 patients. Considering a dropout rate of 10%, a total of 138 patients need to be allocated to reach the required number of patients for the efficacy analysis. For convenience of randomization, we decided to recruit 140 patients. The formula used to calculate the sample size is as follows:

$$\text{n1}={\left(\text{U}\alpha +\text{U}\beta \right)}^{2}\times 2P\times (1-P)/{\left(P1-P2\right)}^{2}=\text{n2}$$

### 2.8 Outcome measures

#### 2.8.1 Background information

Background information includes demographic data and general clinical data. Demographic data consists of gender, age, height, weight, and so on. General clinical data consists of medical history, course of disease, treatment history, combined diseases, concomitant medications, and so on. The participants’ information and privacy will be strictly protected and forbidden to the public.

#### 2.8.2 Safety outcomes

Safety is assessed by vital signs, laboratory examinations, and AEs. Vital signs include body temperature, breathing, blood pressure, and heart rate. Laboratory examinations include blood, urine and stool routine, liver, and kidney function. AEs will be recorded all the time during the treatment. The development of AEs will also be observed until the adverse reactions disappear.

#### 2.8.3 Primary and secondary outcomes

The primary outcome measures of the study are NT-proBNP and left ventricular ejection fraction, which will be measured at baseline and 8 weeks after randomization. Secondary outcomes include plasma levels of microRNA-145, plasma creatine kinase-MB, and CardiaC Troponin I levels in blood samples. At the same time, the trial also observes the occurrence of MACEs defined as the composites of deaths from any cardiac causes, myocardial infarction, and revascularization (PCI or CABG) at 8 weeks after randomization. The efficacy of angina symptoms, Seattle Angina Questionnaire, will be recorded at baseline and 8 weeks after randomization and TCM syndrome efficacy.^[24]^ Items to be measured and the time window of data collection are shown in Table 3.

**Table 3:** Schedule of data collection.

Items	Screening period within 1 wk	Treatment period 4–8 wk	Follow-up period 2–12 mo
Signed informed consent	√		
Inclusion/exclusion criteria	√		
Demographic data	√		
Medical history, course of disease, treatment history	√		
Combined diseases	√		
Concomitant medications	√	√	
LVEF	√	√	
NT-proBNP	√	√	
microRNA-145	√		
CK-MB	√		
cTnI	√		
MACEs			√
SAQ	√	√	
TCM syndrome efficacy	√	√	
Vital signs	√	√	
Blood, urine and stool routine	√	√	
Liver and kidney function	√	√	
Adverse events		√	√

Vital signs: temperature, heart rates, breathing, and blood pressure.

CK-MB = creatine kinase-MB, cTnI = CardiaC Troponin I, LVEF = left ventricular ejection fraction, MACE = major adverse cardiac event, SAQ = Seattle Angina Questionnaire, TCM = Traditional Chinese Medicine.

### 2.9 Adverse events

AEs are defined as negative or unintended clinical manifestations following the treatment. Patients will be asked to report to the investigators any abnormal reactions occurring at any time during the trial. In addition, investigators will collect information about abnormal reactions monthly. All details of related and unexpected AEs, such as time of occurrence, degree and duration of AEs, suspected causes, and the effective measures and outcomes will be recorded on electronic case report form. Any AEs, such as subjective discomfort and laboratory abnormalities, should be taken seriously. Careful analysis and immediate measures are taken to protect the safety of the subjects until the adverse events disappeared.

### 2.10 Quality control of data

Patients’ baseline characteristics are collected with case report forms (CRFs) during hospitalization at least 24 hours after PCI by investigators of each center. Angiographic data are recorded in the PCI procedure. According to the follow-up plan, patients are required to undergo recording of history and echocardiogram in each center at 1- and 12-month follow-up.

Data usage adheres to local laws and regulations of participating centers. Patient privacy is protected by restricting the access to dataset to relevant individuals—investigators, statistical analyzers, clinical research associates, and representatives of the ethics committee—and replacement of the actual names in the documents with serial numbers. Representatives of participating centers are responsible for collecting data of enrolled patients in that center and uploading to the database. Angiographic and echocardiographic data are stored in CD-ROMs and sent to a core lab in Dongfang Hospital, where the data are analyzed by specialists blinded to random allocation.

### 2.11 Statistical analyses

The statistical analyses of primary and secondary outcomes will be conducted in both the intention-to-treat set and the per-protocol set. Primary and secondary endpoint data will be collected for the entire follow-up period for all patients. Patients lost to follow-up will be considered at risk until the date of last contact, at which point they will be censored. In the per-protocol set, patients who undergo randomization but withdraw consent to participate or not treated according to the allocated procedure after randomization will be excluded from per-protocol statistical analyses.

IBM SPSS V17.0 software pack was used for data analysis. The normal distribution of the data was tested with Pearson test. Independent sample *t* test was used for the comparison of data with normal distribution, while Spearman test was used for the comparison of data without normal distribution. Data with normal distribution were expressed as arithmetic mean ± standard deviation, while data without normal distribution were expressed as median value (minimum–maximum). A *P* value between .1 and .05 was considered marginally significant, a *P* value between .01 and .05 was considered significant, .001 and .01 highly significant, and < .001 very highly significant.

### 2.12 Ethics and dissemination

This trial has been registered at Chinese Clinical Trial Registry http://www.chictr.org.cn/showproj.aspx?proj=61713. The data of this trial will be managed by ResMan at http://www.medresman.org/ and posted on Chinese Clinical Trial Registry. The results of this study will be disseminated to the public through academic conferences and peer-reviewed journals.

## 3 Discussion

Several cardioprotective therapies, which have been conferred robust cardioprotection in experimental animal models of acute ischemia and reperfusion injury, have failed in the clinical setting of AMI for patient benefit.^[25]^ Although the cardioprotective strategies act through common end-effectors, multiple mechanisms affect cardiomyocyte death. The treatments emerging from experimental studies may be suboptimal in patients with comprehensive comorbidities.^[26,27]^ Therefore, additive or synergistic cardioprotective effects of multiple cardioprotective agents might be better cardioprotective strategies to reduce myocardial infarction.^[28]^ As a supplementary and complementary medicine, the characteristics of CHM that is multiple treatment target are attracting more attention. As the principal active components, pharmacological studies have shown that Astragalus membranaceus could perform the function of anti-oxidation, regulation of mitophagy, anti-inflammation, immunomodulatory effects, anti-neurodegeneration.^[29,30]^ Salvia miltiorrhiza is reported to protect vascular endothelial cells against hypoxia, regulate proliferation and migration of VSMCs, improve microcirculatory, and have function of anticoagulant, antithrombotic, anti-inflammatory.^[31]^ Previous studies have shown that Cortex Moutan and Lonicerae japonicae flos both have cardioprotective function^[32,33]^ (Table 1). However, whether the postoperative administration of YLS in AMI patients treated with PPCI will improve cardiac function still requires confirmation by randomized, double-blinded, placebo-controlled, parallel-group clinical trial with the objective of determining the effectiveness and safety of YLS.

This trial tried to incorporate miRNA-145 as one of surrogates for hard clinical endpoints. miRNAs are single-stranded RNA molecules 20 to 25 nucleotides in length coded by genes transcribed by DNA, which are known as “noncoding RNA.” While miRNAs are not converted to protein products, they act to gene expression, primarily through translation repression or RNA degradation.^[34,35]^ Several investigations have demonstrated that miRNAs may play a major role in regulating most of the human genome, including gene alterations associated with various aspects of heart disease, such as ischemic MIR.^[36]^ Genetic or pharmacological manipulation of miRNAs has been indicated to modulate the sensitivity of the myocardium to MIR,^[35]^ protective noncoding RNAs may have a role in the setting of anticipated ischemia.^[37]^ Because of their significant role in all stages of MIR, regulating miRNAs is a promising therapeutic strategy. The miRNAs in plasma and serum are reported that are present in a remarkably stable form and can be detected in the peripheral circulation.^[38]^ Therefore, the miRNAs can be considered as promising novel biomarkers for the diagnosis and prognosis of cardiovascular diseases.^[39]^ Previous studies reported that the expression of miRNA-145 is associated with MIR.^[40]^ In animal model of ischemia/reperfusion injury and myocardial hypoxia/reoxygenation-induced cell model, miRNA-145 were significantly reduced,^[41,42]^ which indicated that the abnormal expression of miR-145 may be the good candidate for MIR.

A potential limitation of our study is that the trial will be undertaken in China, it is uncertain whether the similar effects are available to other ethnic groups and regions. To conclude, the aim of this trial is to demonstrate that YLS will protect the myocardial function in patients with AMI after successful PCI and subsequently yield long-term benefit.

## Author contributions

DL and QL are the principal investigator of this study. CC and JYX conceptualized the study design and wrote the manuscript. JW and KZ modified the manuscript. CC, RLF and JYX participated in the establishment of the eCRF. CC, RLF and JYX participated in the recruitment of patients. CC and JYX designed the method for statistic analysis. CC, RLF and JYX will participate in the data collection and analysis. All authors read and approved the final manuscript.

**Conceptualization:** Cong Chen, Dong Li.

**Data curation:** Jie Wan.

**Methodology:** JunYan Xia.

**Software:** Kun Zhou.

**Writing – original draft:** Ruli Feng.

**Writing – review & editing:** Qian Lin.

## Corrections

The corresponding author originally appeared incorrectly as Cong Chen and has since been corrected to Dong Li.
